# Ciprofloxacin-induced Hepatotoxicity in a Healthy Young Adult

**DOI:** 10.7759/cureus.1016

**Published:** 2017-02-08

**Authors:** Zulfiqar Qutrio Baloch, Muhammad Ali Raza, Shabber A Abbas, Sumera Bukhari

**Affiliations:** 1 Internal Medicine, Brandon Regional Hospital; 2 Internal Medicine, Wayne State University, Detroit, MI 48202; 3 Internal Medicine, R-Endocrinology, Hamilton, NJ; 4 Internal Medicine, Seton Hall University-St. Francis Medical Center, Trenton, NJ

**Keywords:** ciprofloxacin, fluoroquinolone, acute hepatitis, drug-induced hepatitis, hepatotoxicity, abdominal pain

## Abstract

Ciprofloxacin is a broad-spectrum fluoroquinolone antibiotic that is widely used in the treatment of many common infections, including urinary tract infections (UTIs). Despite the increase in Escherichia coli resistance to ciprofloxacin, especially in the United States (US), clinicians continue to utilize the high bioavailability of this drug in urine to counter UTIs. A rare adverse effect following use of ciprofloxacin is drug-induced hepatitis. In this case report, we describe a young 29-year-old female with a previous medical history significant for pyelonephritis and ovarian cyst who presented to the emergency room with signs and symptoms suggestive of progressive liver injury for two weeks that started two days after a complete course of ciprofloxacin therapy for a UTI. An extensive workup failed to identify a particular cause for the hepatotoxicity. The associated onset of symptoms following ciprofloxacin use, the pattern of hepatic enzyme elevation coupled with abdominal pain suggestive of liver pathology, and the resolution of all symptoms following supportive therapy all pointed towards the possible diagnosis of ciprofloxacin-induced hepatotoxicity. The patient was treated with supportive therapy, and subsequently, her symptoms resolved over the next few days with the improvement of her liver enzyme levels. The patient was discharged with instructions to avoid ciprofloxacin and other fluoroquinolones in the future. Clinicians should maintain a high degree of suspicion when treating patients with ciprofloxacin who subsequently develop signs and/or symptoms of liver injury.

## Introduction

Ciprofloxacin is a widely prescribed fluoroquinolone that has broad antimicrobial coverage and high oral bioavailability [1]. Severe adverse effects associated with its use include tendon rupture, Stevens-Johnson syndrome, interstitial nephritis, and liver injury [2-3]. Most commonly, liver damage related to ciprofloxacin is limited to an asymptomatic elevation in liver enzymes. In rare instances, it can also present as acute hepatitis. Here, we describe such a case of acute hepatitis following a short course of ciprofloxacin therapy.

## Case presentation

A 29-year-old female with a past medical history of pyelonephritis and an ovarian cyst presented to the emergency room with the complaint of right upper quadrant abdominal pain radiating to the epigastrium for two weeks. She described the pain as a dull, aching pain with the intensity of 3 out of 10 on the Numeric Pain Scale. The pain was progressive and constant with no significant aggravating or relieving factors. The pain was accompanied by nausea but without vomiting. She denied any fever, chills, rash, diarrhea, vaginal discharge, or dysuria at the time of presentation. The patient had no significant past surgical history, and family history was non-contributory. She denied smoking or the use of alcohol or any illicit drug. The patient reported an allergy to codeine, which causes hives. One week before the onset of her symptoms, she reported an episode of dysuria with hematuria and was diagnosed by her primary care physician with a urinary tract infection (UTI). She was treated with oral ciprofloxacin, 500 mg once a day for five days. Her UTI symptoms subsequently resolved. She denied taking any other medications. 

Pertinent findings during physical examination included abdominal tenderness in the right upper and lower quadrants associated with guarding in the right lower quadrant. The rest of the physical examination was unremarkable. Laboratory studies showed some abnormal values as mentioned: total bilirubin - 1.5 mg/dL (reference range: 0.1 - 1.0 mg/dL), direct bilirubin - 0.20 mg/dL (reference range: 0.0 - 0.3 mg/dL), serum aspartate aminotransferase (AST) - 354 U/L (reference range: 8 - 20 U/L), serum alanine aminotransferase (ALT) - 766 units/L (reference range: 8 - 20 U/L ), and alkaline phosphatase (ALP) - 159 units/L (reference range: 50 - 100 U/L). Other metabolic and hematological parameters were normal on workup. A urinalysis did not show any significant abnormality, and a pregnancy test was negative. A complete follow-up investigation was conducted to ascertain the cause of acute hepatitis. Viral hepatitis serologies, toxicology screen, and autoimmune markers were all normal. Computed tomography (CT) of the abdomen and pelvis with contrast was done in the emergency room and it showed a 3.8 cm enhancing mass within the right lobe of the liver. Correlation with magnetic resonance imaging (MRI) scan was recommended. Contrast MRI of the abdomen and pelvis demonstrated the right hepatic lesion most likely to be focal nodular hyperplasia, and follow-up contrast-enhanced MRI in two to three months was recommended. Further, biopsy of the lesion was not opted due to the benign nature of the lesion. However, the patient was advised on the discharge to get a follow-up MRI. 

It was suggestive that the liver injury was secondary to use of ciprofloxacin since the symptoms started two days following the completion of the antibiotics for the treatment for UTI. Given the associated onset of symptoms following ciprofloxacin use, the pattern of hepatic enzyme elevation coupled with abdominal pain suggestive of liver pathology, and the resolution of all symptoms following supportive therapy all pointed towards the diagnosis of ciprofloxacin-induced acute hepatitis. The patient was treated with supportive therapy with hydration and anti-emetics as needed. Subsequently, the patient's symptoms resolved over the next few days with the improvement of her liver enzyme levels. 

## Discussion

There is a broad spectrum of drug-induced liver diseases ranging from an asymptomatic elevation in liver enzymes and hepatitis to fulminant hepatic failure. Drug-induced hepatitis may account for approximately 10 to 50% of all cases of elevated liver enzymes, while up to 25% of fulminant hepatitis may be associated with the use of medication [3]. Drug-induced hepatotoxicity may present with various systemic manifestations, may present as acute autoimmune hepatitis, can evolve into cirrhosis, and can also mimic veno-occlusive disorders. Having a high index of suspicion is important for clinicians to recognize and discontinue any medication suspected of producing such reactions.

Fluorinated quinolones, including ciprofloxacin, are metabolized in the liver and excreted by the kidneys. Ciprofloxacin has been shown to cause a 1% to 3% self-resolving transient elevation in liver enzymes. Patients who present with ciprofloxacin-induced acute hepatitis have similar symptoms to those with nondrug-induced acute hepatitis. These include right upper quadrant abdominal pain, malaise, anorexia, low-grade fever, nausea, vomiting, and dark urine. Severe cases may also exhibit coagulopathy and/or encephalopathy. Hepatomegaly and jaundice may be prominent on physical examination [4]. There are no established risk factors for ciprofloxacin-induced hepatitis; however, certain factors can be associated with it. Elderly patients are more at risk of ciprofloxacin-induced hepatotoxicity with the risk increasing if hepatotoxic drugs or alcohol are being used concomitantly. The presence of underlying liver diseases, such as hepatitis B or C, can also be an additional risk in itself. Certain genes that control the transport, metabolism, and detoxification of fluoroquinolones also play their role in a drug-induced liver injury.

Ciprofloxacin at times causes acute liver injury within a period of two days to two weeks following the initiation of antibiotic treatment. Although the precise mechanism of ciprofloxacin-induced liver injury remains currently unknown, hepatocellular necrosis leading to elevated liver enzymes has been observed. The pattern of injury can be cholestatic, hepatocellular, or mixed. The more familiar pattern in the acute setting, such as in our patient, is hepatocellular, which is associated with markedly elevated alanine transferase levels. The cholestatic pattern of liver injury usually occurs after a prolonged course of antibiotic use [4]. The mechanism of hepatic damage caused by drugs and toxins is either due to their direct toxic effect or an idiosyncratic reaction. The direct toxic effect is dose-dependent while idiosyncratic reactions can be metabolic or immune-mediated and can occur at any dosage of the drug. In most cases, it is likely an idiosyncratic hypersensitivity response because of the short latency period, frequent immuno-allergic features, and severe disease upon re-exposure [5]. The type of injury caused by ciprofloxacin and other drugs can be differentiated by the level of liver enzymes elevated. If the ALP is elevated more than two times its upper normal limit or the ALT/ALP ration is less than 2, this indicates cholestatic hepatitis. If the ALT/ALP ratio is between 2 and 5, then it is likely a mixed pattern. If the ratio is more than 5, this demonstrates hepatocellular injury. The prognosis is worse if the patient has elevated bilirubin levels more than two times its upper limit in association with elevated serum aminotransferases [6-7].

There are no specific lab tests or histological findings that can establish ciprofloxacin as the definite cause of drug-induced hepatitis. Any development of hepatitis or symptoms indicative of liver injury following ciprofloxacin use should prompt immediate discontinuation and further investigation and evaluation. Given the lack of specific diagnostic findings in general, the diagnosis of ciprofloxacin-induced hepatotoxicity should be a diagnosis of exclusion. It involves thorough history taking and physical examination, as well as a complete liver function and hepatitis panels to rule out other more common causes of acute hepatitis. In all cases, it is necessary to inquire about any previous adverse event with the drug and alleviation of symptoms with drug discontinuation and supportive therapy [8].

Fluoroquinolone-induced hepatitis is usually nonfatal and is self-resolving after drug discontinuation and symptomatic treatment. Only four previous reports of ciprofloxacin-induced liver injury, including that of our patient's case, have been reported in the literature [9]. There has been at least one fatality reported with the ciprofloxacin-induced liver injury in a 74-year-old woman undergoing treatment for UTI. In her case, symptoms began following the use of ciprofloxacin; however, she was given a second course of the drug due to a non-resolving UTI. Serial monitoring of liver enzymes should be followed until they return to normal. Corticosteroids might be used with variable success, although their use was not warranted in our particular case. Patients with prominent features of hypersensitivity may find corticosteroids more beneficial in alleviating symptoms. Given the propensity of ciprofloxacin-induced acute hepatitis being a class effect, patients who develop drug-induced hepatitis following ciprofloxacin use should be advised to avoid further use not only of ciprofloxacin but fluoroquinolones in general as well.

## Conclusions

Ciprofloxacin-induced acute hepatitis is often an idiosyncratic reaction causing hepatocellular necrosis. Other factors may also play their role in hepatotoxicities, such as age, genetic factors, and concomitant drug or alcohol use. Patient developing liver injury with any fluoroquinolone should be advised to avoid other antibiotics from the same group because of their cross-sensitivity.
